# External validation of the T.O.HO. score as predictor of success after retrograde intrarenal surgery

**DOI:** 10.1186/s12894-022-01018-3

**Published:** 2022-04-24

**Authors:** Samet Senel, Yusuf Kasap, Yalcin Kizilkan, Sedat Tastemur, Cuneyt Ozden

**Affiliations:** Department of Urology, Ankara City Hospital, Üniversiteler, Bilkent Blv. No: 1, 06800 Çankaya, Ankara Turkey

**Keywords:** RIRS, Stone scoring system, T.O.HO. score

## Abstract

**Background:**

To assess the effectiveness of T.O.HO. (Tallness, Occupied lesion, Houndsfield unit evaluation) score in predicting the retrograde intrarenal surgery (RIRS) success and to validate this scoring system.

**Methods:**

The age, sex, previous stone surgery, hospitalization, surgery duration, postoperative complication, stone length, stone location, stone density, stone number, lateralization, presence of hydronephrosis, and presence of preoperative stent datas of 611 patients who underwent RIRS in our clinic between January 2013 and January 2021 were retrospectively assessed. The patients were divided into two groups as successful and unsuccessful. The T.O.HO scores of all patients were calculated.

**Results:**

The success rate was 72.5%. Compared to the unsuccessful group, stone length and stone density were lower, surgery duration was shorter and there were less lower pole stones in the successful group (p < 0.001). No significant difference was found between the two groups in terms of the other parameters. The T.O.HO. score was significantly lower in the successful group compared to the unsuccessful group (p < 0.001). According to the multivariate logistic regression analysis, stone length (OR: 0.905; 95% Cl: 0.866–0.946; p < 0.001), lower pole location (OR: 0.546; 95% Cl: 0.013–0.296; p < 0.001), stone density (OR: 0.999; 95% Cl: 0.998–1; p = 0.044) and the T.O.HO. score (OR: 0.684; 95%Cl: 0.554–0.844; p < 0.001) were found as the independent risk factors for RIRS success. ROC curve analysis showed that the T.O.HO. score could predict the RIRS success with 7.5 cut-off point (AUC: 0.799, CI: 0.76–0.839; p < 0.001).

**Conclusion:**

The T.O.HO. score can predict RIRS success with a high rate of accuracy.

## Background

Urinary system stone disease is a significant health problem, the frequency of which is gradually increasing and impairs the quality of life [1]. It is known that higher ambient temperature has an association with urinary system stone disease [2]. Risk of lifelong urinary system stone disease is 13% in men and 7% in women in the general population [3]. Also in recent studies, it was shown that the rise in prevalence of urinary system stone disease is greater among women than men [4]. Retrograde intrarenal surgery (RIRS) has become a preferred method by the patients and surgeons with its advantages of low morbidity and not requiring incision [5]. The studies have revealed that the success rate of RIRS was 50–94.2%. It was shown that a stone-free rate of 91% could be achieved with more than one procedure even in large kidney stones [6–8]. However, it is necessary to choose the appropriate surgical method in order to achieve a good success rate,

Various scoring systems were developed for determining the appropriate surgical method and increasing success rate [9–11]. Besides, a new stone scoring system—the T.O.HO. (Tallness, Occupied lesion, Houndsfield unit evaluation) score- was defined in 2020 by Hori et al.. In this scoring system, the parameters of stone length, stone localization and stone density are assessed and patients are scored between 3 and 11 points. Higher score is associated with low success [12]. Even though it was shown in this study that the T.O.HO. score was an effective scoring system in predicting RIRS success, external validations in with a larger number of procedures is necessary to confirm. The purpose of this study is to validate the T.O.HO. score.

## Methods

**Fig. 1:**
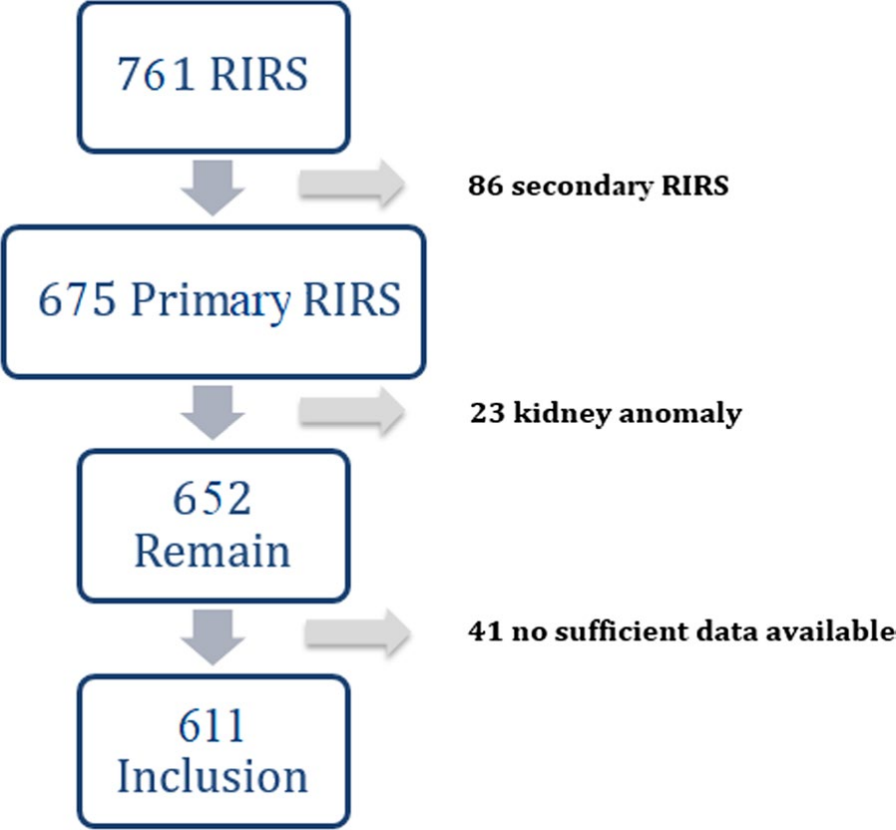
Flowchart of included patients

The present study was prepared in accordance with the principles of Helsinki Declaration. The No 2 Clinical Research Ethics Committee of Ankara City Hospital approved this retrospective study and waived informed consent. (Ethics Committee approval number: E2-21-351). In our tertiary hospital, the datas of 761 patients who underwent primary RIRS between January 2013 and January 2021 due to kidney and proximal ureter stones were retrospectively analyzed. 86 patients operated with secondary RIRS, 23 patients having kidney anomaly and 41 patients not having sufficient data were excluded from the study. A total of 611 RIRS cases were included in this single centre study (Fig. 1).

The age, sex, hospitalization, surgery duration data of all patients were obtained from the hospital information database and retrospectively assessed. Diagnoses of all patients were established through preoperative computerized tomography (CT). According to CT, stone length (mm), stone location (ureter and pelvis, middle and upper pole, lower pole), stone density (HU [Houndsfield Unit]), stone number (single-multiple), lateralization (right-left), presence of hydronephrosis and presence of preoperative stent were assessed. As it was defined in the T.O.HO. score, the size of the stones was determined by measuring the longest axis in the preoperative radiological analysis. In multiple stone cases, the size of the stones was defined as the two-dimensional area determined by multiplying the longest diameter by the perpendicular diameter of the stones. On the other hand, stone density was determined as the average density of the stones. As it was described in the T.O.HO. score, patients were scored between 3 and 11 points by using the parameters of stone length (< 10 mm: 1 point, 10–17 mm: 2 points, 17–24 mm: 3 points, 24–30 mm: 4 points, > 30 mm: 5 points), stone location (proximal ureter and renal pelvis: 1 point, middle and upper pole: 2 points, lower pole: 3 points), and stone density (< 620 HU: 1 point, 620–1100 HU: 2 points, > 1100 HU: 3 points) [12]. Two independent authors (SS and ST) assessed the parameters the T.O.HO. score and calculated the score between September 2021-October 2021. A third independent author (YK) resolved any disagreement.

Patients having positive urine culture were treated with appropriate antibiotics for at least 7 days in the preoperative period. Prophylaxis with intravenous 2 g of cefazolin was administered to all patients within 1 h before surgery. All cases were performed under general anesthesia and in lithotomy position. Ureterorenoscopy was performed before RIRS with 9.5 F rigid renoscope for dilatation (Karl Storz, Tuttlingen, Germany). In cases where the ureteral dilatation was sufficient, collecting system was approached through access sheath. Following this, proximal ureter and kidney were reached by entering from access sheath with 7.5 F flexible ureterorenoscope (Karl Storz, Flex X2, GmbH, Tuttlingen, Germany). On the other hand, in cases where the access sheath could not reach proximal ureter and kidney due to insufficient ureteral dilatation, the operation was postponed to two weeks later by placing a double J (DJ) stent. The stones was fragmented with fragmentation method with the energy of 1.5–2 J, the frequency of 5 Hz and power of 7.5–10 W by using a holmium-yttrium–aluminum-garnet (Ho:YAG) laser (200 μm) that conveyed in the working channel of the flexible ureterorenoscope. A 1.9 F nitinol basket catheter was used for extraction of the fragmented stones. At the end of the operation, an ureteral stent and urethral catheter were inserted in all patients. All the operations were performed by three surgeons having an experience of at least 10 years. Patients were assessed with CT on the postoperative first month. Success was defined as the absence of stones in the urinary tract. Accordingly, patients were divided into two groups as successful and unsuccessful.

### Statistical analysis

The coding and statistical analyses of the data were made on the computer using SPSS 22 software (IBM SPSS Statistics, IBM Corporation, Chicago, IL) package program. The compliance of the variables to the normal distribution was analyzed through Shapiro–Wilk tests. Mann–Whitney U test was used in comparing the non-categorical parameters among the groups. For categorical variables, chi-square or Fisher’s exact tests were used. Backward LR method and multivariate logistic regression analysis were used in assessing the parameters considered to be likely an independent risk factor for RIRS success. The predictive characteristic of the T.O.HO. score for RIRS success was analyzed with ROC curve with a confidence interval of 95%. Cases with p value of < 0.05 were accepted as statistically significant.

## Results

611 patients were included in the study. The mean age of the patients was 46.1 ± 14.2 years. Mean stone length was 16.3 ± 8.4 mm. Success rate was 72.5%. No difference was detected between successful and unsuccessful groups in terms of age, sex, stone number, lateralization, previous stone surgery, presence of preoperative stent, presence of hydronephrosis, postoperative complication, and hospitalization (respectively p = 0.209, p = 0.671, p = 0.097, p = 0.999, p = 0.163, p = 0.218, p = 0.062, p = 0.199, and p = 0.296). Stone length and density were lower and surgical duration was shorter in the successful group in a statistically significant manner (p < 0.001). On the other hand, the unsuccessful group had more lower pole stones than the successful group (% 61.9 vs % 37, p < 0.001). Compared to the unsuccessful group, the T.O.HO. score was significantly lower in the successful group (p < 0.001). Table 1 shows demographic, clinical characteristics and perioperative variables of the patients. It was observed that as T.O.HO. score of the patients increased, their RIRS success rate decreased (p < 0.001) (Table 2). According to the multivariate logistic regression analysis, stone length (OR: 0.905; 95% Cl: 0.866–0.946; p < 0.001), lower pole location (OR: 0.546; 95% Cl: 0.013–0.296; p < 0.001), stone density (OR: 0.999; 95% Cl: 0.998–1; p = 0.044) and the T.O.HO. score (OR: 0.684; 95% Cl: 0.554–0.844; p < 0.001) were found to be the independent risk factors for RIRS success (Table 3).

**Table 1:** Demographic, clinical characteristics and perioperative variables of patients who underwent RIRS for lower pole kidney stones

	Total (n = 611)	Successful (n = 443, 72.5%)	Unsuccessful (n = 168, 27.5%)	p
Age (years)	46.1 ± 14.2	45.7 ± 14.3	47.1 ± 13.8	0.209^ m^
Sex				0.671 ^c^
Male, n (%)	385 (63)	277 (62.5)	108 (64.4)	
Female, n (%)	226 (37)	166 (37.5)	60 (35.6)	
Stone length (mm) (mean ± SD)	16.3 ± 8.4	14 ± 5.5	22.5 ± 11.2	** < 0.001 **^m^
Stone location				** < 0.001 **^c^
Ureter and renal pelvis, n (%)	251 (41.1)	201 (45.4)	50 (30)	
Middle and upper pole, n (%)	91 (15)	78 (17.6)	14 (8.1)	
Lower pole, n (%)	269 (43.9)	162 (37)	104 (61.9)	
Stone density (HU) (mean ± SD)	962.1 ± 327.2	914.5 ± 321	1087.3 ± 310.6	** < 0.001** ^m^
Stone number				0.097 ^c^
Single, n (%)	395 (64.7)	295 (66.7)	100 (59.4)	
Multiple, n (%)	216 (35.3)	148 (33.3)	68 (40.6)	
Lateralization				0.999 ^c^
Right, n (%)	290 (47.5)	210 (47.5)	80 (47.5)	
Left, n (%)	321 (52.5)	233 (52.5)	88 (52.5)	
Precence of preoperative stent				0.218 ^c^
Yes, n (%)	99 (16.2)	77 (17.3)	22 (13.1)	
No, n (%)	512 (83.8)	366 (82.7)	146 (86.9)	
Precence of hydronephrosis				0.062 ^c^
Yes, n (%)	358 (58.7)	270 (61)	88 (52.5)	
No, n (%)	253 (41.3)	173 (39)	80 (47.5)	
Surgical duration (min) (mean ± SD)	54.3 ± 18.3	50.6 ± 16.6	64 ± 19.1	** < 0.001** ^m^
Hospitalization (day) (median) (min–max)	1(1–15)	1(1–10)	1(1–15)	0.296 ^m^
T.O.HO. score (mean ± SD)	6.6 ± 1.9	6 ± 1.7	8.1 ± 1.5	** < 0.001** ^m^

Bold *p* values ​​indicate statistical significance

*RIRS* Retrograd Intrarenal Surgery, *HU* Houndsfield Unit, *SD* standard deviation, *T.O.HO.* (T)allness, (O)ccupied Lession Status, (HO)undsfield Unit Evaluation, ^*m*^ Mann Whitney U Test, ^*c*^ Chi-square Test

**Table 2:** Success rate after RIRS according to the T.O.HO. score

T.O.HO. score	Success rate, % (n)
3	98.2% (55/56)
4	91.7% (11/12)
5	91.5% (86/94)
6	87.2% (95/109)
7	73.6% (114/155)
8	57.9% (55/95)
9	32.8% (19/58)
10	27.3% (6/22)
11	20% (2/10)
Total	72.5% (443/611)
	**p < 0.001**^c^

Bold *p* value ​​indicates statistical significance

*RIRS* Retrograd Intrarenal Surgery, *T.O.HO.* (T)allness, (O)ccupied Lession Status, (HO)undsfield Unit Evaluation, ^***c***^ Chi-square test

**Table 3:** Multivariate logistic regression analysis of potential independent risk factors for postoperative success rate

Parameters	OR (95% CI)	p
Stone length (mm)	0.905 (0.866–0.946)	** < 0.001**
Lower pole location	0.546 (0.013–0.296)	** < 0.001**
Stone density (HU)	0.999 (0.998–1)	** 0.044**
T.O.HO. score	0.684 (0.554–0.844)	** < 0.001**

Bold *p* values ​​indicate statistical significance

*HU* Hundsfield Unit, *T.O.HO.* (T)allness, (O)ccupied Lession Status, (HO)undsfield Unit Evaluation

**Fig. 2:**
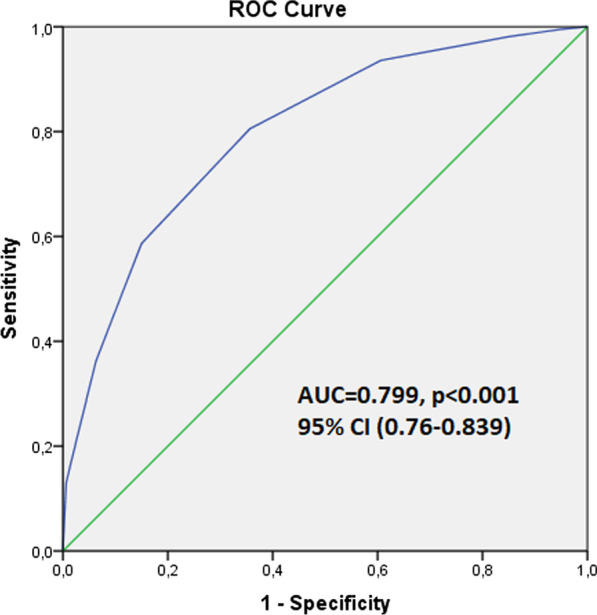
ROC curves for predicting success of retrograde intrarenal surgery based on T.O.HO. score

**Fig. 3:**
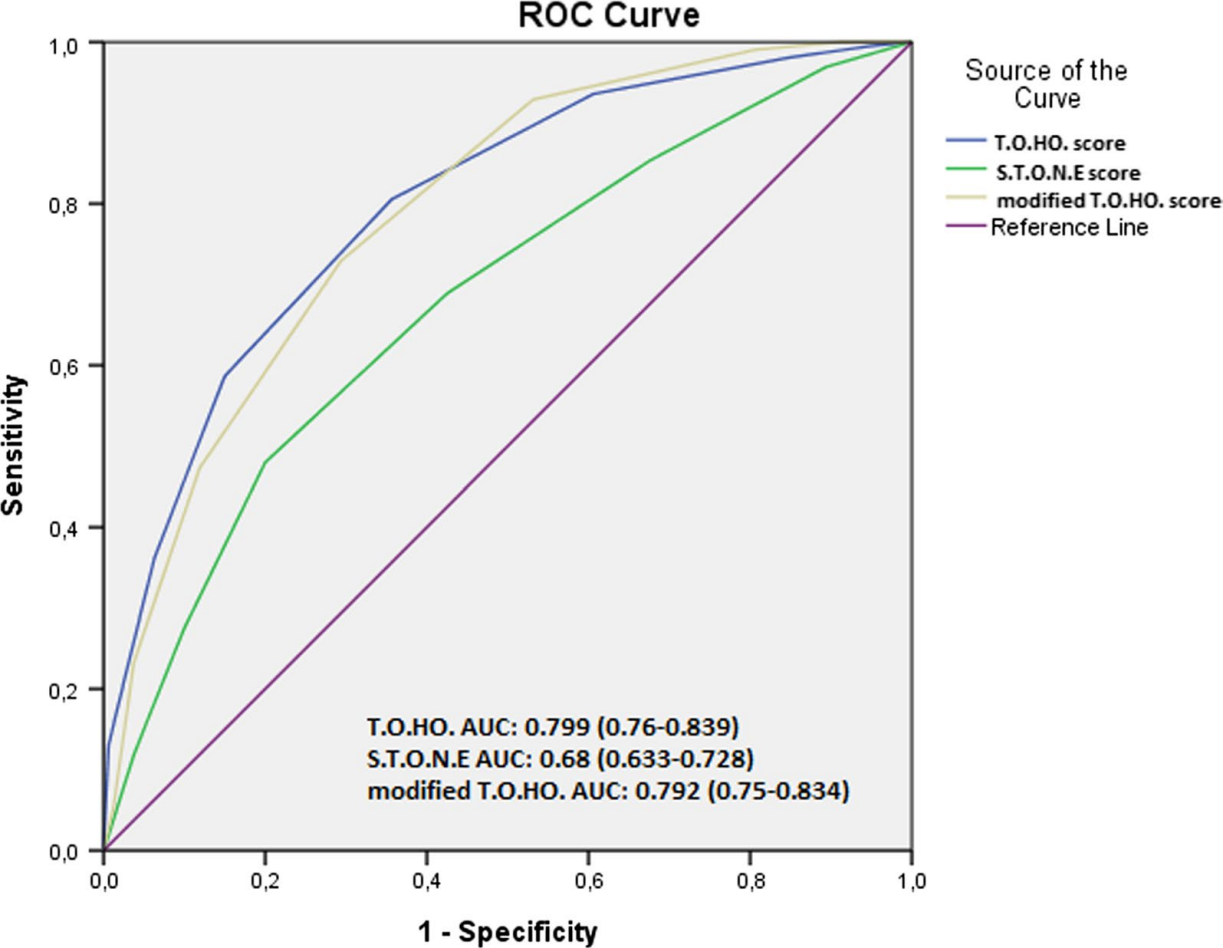
ROC analysis of the original T.O.HO., STONE, and modified T.O.HO., in predicting retrograde intrarenal surgery failure

Afterwards, ROC curves were generated for the purpose of assessing the effectiveness of the T.O.HO. score in predicting the success in RIRS. The AUC of T.O.HO. score was 0.799 with an optimal cut-off value of 7.5 point, which showed a sensitivity of 0.805 and specificity of 0.761 (95% CI 0.76–0.839) (Fig. 2). Also, the effectiveness of the original T.O.HO. score defined by Hori et al. [12], S.T.O.N.E score defined by Molina et al. [11], and modified T.O.HO. score defined by Polat et al. [13] in predicting RIRS success was evaluated by ROC analysis by using our datas. Among these scoring systems, T.O.HO. score and modified T.O.HO. score had similar effectiveness but higher predictive power than S.T.O.N.E score (Fig. 3).

## Discussion

The primary purpose of the treatment of urinary system stone disease is to provide minimal morbidity and maximum stone-free rate. The treatment of kidney stones significantly changed in the last 30 years. The treatment options have diversified as percutaneous nephrolithotomy (PNL), shock wave lithotripsy (SWL), and RIRS, which are less invasive than open surgery [14]. The use of RIRS particularly has been increasing in the treatment of upper urinary system stones day by day. Having less complication rates compared to PNL and having higher stone-free rates compared to SWL are the most significant reasons of why this method is frequently used [15, 16]. Flexible ureterorenoscope is an expensive equipment. Therefore, it should be used effectively. Besides, the results should be predictable in order to meet the expectations of the patients and plan the surgical method [17].

With the development of minimal invasive surgical techniques, scoring systems were produced to predict the success and complication rates. For RIRS, Resorlu-Unsal stone score (RUSS) predicting the stone-free rate was defined by Resorlu et al., for the first time in literature in 2012. In RUSS, the parameters of stone size, stone location and infundibulopelvic angle (IPA), stone number and presence of abnormal anatomy are assessed [9]. In another scoring system, the R.I.R.S. scoring system, the parameters are stone density, IPA, infundibular length (IL), stone length and stone localization [10]. On the other hand, in S.T.O.N.E. (Stone size, Tract length, Obstruction, Number of involved calyces and Essence or stone density) nephrolithometry scoring system, the parameters of stone size, number of involved calyces, tract length, obstruction/hydronephrosis and stone essence are assessed. Different from the others, the T.O.HO. score can be used for both ureter and kidney stones [11]. In all the scoring systems, the stone-free rate is decreased with increasing score.

The T.O.HO. score was validated in a recent study by Polat et al. The modified T.O.HO. score was defined by adding stone volume to the parameters of the T.O.HO. score. In this study, modified T.O.HO. score was shown to predict the RIRS success better than the original version [13]. But in our study, T.O.HO. score and modified T.O.HO. score had similar effectiveness in predicting RIRS success.

The T.O.HO score is a simple scoring system defined by Hori et al., in 2020 as a result of the retrospective analysis of 586 patients with kidney and ureter stones who underwent RIRS. It consists of three parameters; stone length (Tallness) (1–5 point), stone localization (Occupied lesion) (1–3 point) and stone density (Houndsfield unit evaluation) (1–3 point). Therefore, the patients are scored between 3 and 11 points. As the score increased, RIRS success decreased. Here, success was defined as having no residue stone in postoperative first month. The stone-free rate was 80.2% [12].

It was concluded in the present study that the T.O.HO. score was easily applicable. It already consists of simple parameters we take into consideration in our clinic practice. All the parameters can easily be assessed through preoperative CT. In this study involving 611 patients, we aimed to assess the findings of Hori et al., and to validate the T.O.HO. score. We revealed that the T.O.HO. score is a stone scoring system that predicts the RIRS success with a high rate of accuracy.

With its some characteristics, the T.O.HO. score has some advantages compared to the other scoring systems. Firstly, it is seen that the predictive quality of the T.O.HO. score is better than S.T.O.N.E score even though it uses fewer parameters than S.T.O.N.E. score in the internal validation carried out by Hori et al., (respectively, AUC = 0.833, AUC = 0.633). Besides, the IPA assessed in RUSS and R.I.R.S. scoring systems can only be assessed through CT urography, whereas non-contrast CT is sufficient in the assessment of T.O.HO. score parameters.

There are several established and verified nomograms and scoring systems in endourology. Although they can help in the decision making, the decision for treatment and surgery should be individualized according to the patient's current condition and expectations [18].

There are several limitations of the present study. These limitations are that the study were designed retrospectively, the present study reflects the results of a single center and the operations performed by 3 surgeons, and the number of patients with preoperative stent was great. Nevertheless, we think that our study validating the T.O.HO score will contribute to the literature.

## Conclusion

This study validates the T.O.HO. score as a stone scoring system predicting RIRS success. The T.O.HO. score is a stone scoring system that is easy to apply and has a high rate of accuracy.

## Data Availability

The datasets generated and/or analysed during the current study are available in Figshare Repository at https://figshare.com/s/2fc947ea33bb8cc14bd4.

## Abbreviations

RIRS: Retrograde intrarenal surgery
T.O.HO.: Tallness, Occupied lesion, Houndsfield unit evaluation
CT: Computerized tomography
HU: Houndsfield Unit
DJ: Double J
Ho:YAG: Holmium-yttrium–aluminum-garnet
PNL: Percutaneous nephrolithotomy
SWL: Shock wave lithotripsy
RUSS: Resorlu-Unsal stone score
IL: Infundibular length
S.T.O.N.E.: Stone size, Tract length, Obstruction, Number of involved calyces and Essence or stone density
